# Preparticipation screening in athletes, the role of sports cardiology and the impact of the camera

**DOI:** 10.1007/s12471-018-1088-2

**Published:** 2018-02-08

**Authors:** R. J. G. Peters

**Affiliations:** Department of Cardiology, F3-326, Academic Medical Center, Amsterdam, The Netherlands

Sports medicine has been receiving increased exposure in communication media for a number of reasons. First, the mass introduction of social media and smartphones has dramatically increased the impact of health-related incidents, in particular cardiac arrests, occurring during sporting events [1]. Such catastrophes are exceedingly rare, but there are countless cameras present at sporting events. Current mass media enable eyewitnesses to distribute the footage instantly. Images of these dramatic events can go ‘viral’ within hours [2].

Second, new screening techniques, such as magnetic resonance imaging (MRI) and genetic testing, have increased our knowledge about abnormalities that may be associated with an increased risk of arrhythmias [3, 4]. These new techniques did increase our ability to identify individuals who are at a high risk, but also introduced new uncertainties. The relevance of new findings is frequently unknown, and the risk of a false positive test result (i. e. an abnormal finding without a real increase in the risk of fatal arrhythmia) is real and present. Abnormal test results associated with potentially life-threatening arrhythmias often imply disqualification for the athlete involved. The impact is tremendous, their career as an athlete may be over.

Third, the availability of new treatments, including implantable defibrillators, has spurred discussions whether or not athletes are able to engage in competitive sports with these devices or with other treatments [5, 6]. Defibrillators do not prevent events, but increase the probability of survival should a cardiac arrest occur. Sports clubs wish to know exactly what the risks are when they hire a professional athlete to play for them. Fourth, the countless cameras that follow sporting events also record the responses by health professionals in case of a suspected or known cardiac arrest [1]. Their skills and behaviour have become the subject of debate and even of legal disputes regarding causality in case of damage or fatal outcome. Fifth, the financial consequences of adverse events for professional athletes and their employers are greater than ever before—the same is true for the liabilities of the physicians who perform preparticipation screening or who assist in accidents on the pitch.

The medical community needs to respond to these developments. New screening techniques should be properly validated before introduction and we need expert analysis of screening tests, including findings from echocardiography and electrocardiography. Although every cardiologist in the world knows how to use these diagnostic tools, the meaning of certain findings in athletes is not necessarily clear. The distinction between pathological changes and physiological adaptation to the participation in high-intensity sports can be challenging. In addition, the quality and quantity of physiological adaptation depends on the type of sporting activities. Assessment therefore requires knowledge of the changes that may be expected from particular sporting activities.

Normal values demonstrated in population studies only partially apply to athletes and the impact of age, gender and, particularly, ethnicity has not been sufficiently documented for use in athletes. Therefore, expert analysis is required in daily diagnostics and in new and complex tests (such as genetic testing and MRI). Especially in genetics, we know little about the prevalence and phenotypic expression of different potentially pathogenic mutations in athletes or about the possible adverse effects of high-intensity training (as has been suggested in hypertrophic cardiomyopathies) [3, 4]. Some mutations may even be associated with improved performance. There is an increasing need for centres of expertise that sports physicians or sports cardiologists can consult to formulate well-informed advice.

Furthermore, sports physicians in training should be exposed to resuscitation scenarios and learn about the different inherited cardiac diseases that are at potential risk of cardiac events—especially cardiomyopathies—and about the implication of cardiogenetics [7]. Medical and paramedical teams assisting at sporting events should be trained in basic life support and in the use of automatic external defibrillators [7]. They should also be periodically retrained and reassessed. These measures will improve the likelihood of a favourable outcome in the rare event of a cardiac arrest in athletes. They may also prevent an unfavourable outcome in terms of the professional reputation of the medical and paramedical teams.

The current special issue of the Netherlands Heart Journal, with contributions from experts in the field, provides valuable information that will increase the quality of preparticipation screening in athletes and that, I trust, the reader will find useful.
